# Soybean and Cotton Spermosphere Soil Microbiome Shows Dominance of Soilborne Copiotrophs

**DOI:** 10.1128/spectrum.00377-23

**Published:** 2023-06-01

**Authors:** Oluwakemisola E. Olofintila, Zachary A. Noel

**Affiliations:** a Department of Entomology and Plant Pathology, Auburn University, Auburn, Alabama, USA; Huazhong Agricultural University

**Keywords:** cotton, microbiome, soybean, spermosphere

## Abstract

The spermosphere is the transient, immediate zone of soil around imbibing and germinating seeds. It represents a habitat where there is contact between seed-associated microbes and soil microbes, but it is studied less than other plant habitats. Previous studies on spermosphere microbiology were primarily culture based or did not sample the spermosphere soil as initially defined in space and time. Thus, the objectives of this study were to develop an efficient strategy to collect spermosphere soils around imbibing soybean and cotton in nonsterile soil and investigate changes in microbial communities. The method employed sufficiently collected spermosphere soil as initially defined in space by constraining the soil sampled with a cork borer and confining the soil to a 12-well microtiter plate. Spermosphere prokaryote composition changed over time and depended on the crop within 6 h after seeds were sown. By 12 to 18 h, crops had unique microbial communities in spermosphere soils. Prokaryote evenness dropped following seed imbibition, with the proliferation of copiotrophic soil bacteria. Due to their long history of plant growth promotion, prokaryote operational taxonomic units (OTUs) in *Bacillus*, *Paenibacillus*, *Burkholderia*, *Massilia*, *Azospirillum*, and Pseudomonas were notable organisms enriched. Fungi and prokaryotes were hub taxa in cotton and soybean spermosphere networks. Additionally, the enriched taxa were not hubs in networks, suggesting that other taxa besides those enriched may be important for spermosphere communities. Overall, this study advances knowledge in the assembly of the plant microbiome early in a plant’s life, which may have plant health implications in more mature plant growth stages.

**IMPORTANCE** The central hypothesis of this research was that plant species and seed exudate release would alter the assembly of microbes in the spermosphere soil. Our research investigated the response of microbes to the initial burst of nutrients into the spermosphere soil, filling knowledge gaps from previous studies that pregerminated seeds under sterile conditions. We identified several copiotrophic bacterial lineages with a long history of plant growth promotion proliferating in response to the initial exudate release. With a comparative network approach, we show that these copiotrophic bacteria are not central to networks, demonstrating that other microbes (including fungi) may be important for community structure. This study improves knowledge on microbial dynamics in the understudied spermosphere and helps inform solutions for biologically or ecologically motivated solutions to spermosphere pathogens.

## INTRODUCTION

When a seed is sown, it imbibes water and releases nutrient-rich exudates that fuel interactions between soil and seed-associated microbes in a plant habitat called the spermosphere (1–5). Seed exudates have long been recognized to stimulate microbial growth, including a direct link to facilitating pathogen growth chemotactically toward seeds (1, 6–8). The spermosphere or “spermatosphere” was first described by Slykhuis (8), who observed the inhibition of a fungal pathogen by three fungal species around a germinating seed. Verona (9) defined the same habitat as a “zone of elevated microbial activity” around a germinating seed. Nelson (1, 5, 10–12) more formally defined the spermosphere as “the short-lived, rapidly changing and microbiologically dynamic zone of soil surrounding a germinating seed,” (1) which is the definition we adhere to here. Despite its importance for plant health outcomes, the spermosphere is less studied than other plant-associated habitats such as the rhizosphere or phyllosphere (3, 4, 13).

Seed germination occurs in three distinct phases. Phase I is a physical process characterized by seed imbibition and fast carbon-rich exudate release into the soil hours after seeds are sown. The highest levels of exudate release are completed in as little as 6 h (1, 14, 15). The initial phase of exudate release is followed by a plateau characterizing phase II and then radical emergence, which begins the formation of the rhizosphere, and more exudate release in phase III (1, 3).

The spermosphere represents a critical zone for establishing vertically inherited seed microbes and horizontal interactions between soil and seed-associated microbes (4, 16–19). The outcome of these interactions can affect the life or death of the plant soon after seeds are sown (5, 20). For example, soilborne *Pythium* can fully colonize and kill germinating seeds of various crop species within 12 to 24 h (10, 11, 21, 22).

Spermosphere pathogens still cause millions of dollars in crop loss yearly (23–25). Because of enhanced genetics and other factors, seed ranks first or second in operating costs borne by soybean and cotton farmers each year (26). Additionally, trends toward earlier planting dates, increased frequency of heavy pulsed rain events, and variable temperature conditions experienced by farmers at planting can create soil moisture and temperature conditions that stress the germinating seed (27–29). Conservation tillage (low or no till) can also lead to harboring of plant pathogens on plant debris left in the field from the previous growing season (30). Consequently, the protection of seeds from pathogens that specialize in spermosphere colonization is vital to improved crop productivity.

Seed and seedling pathogens are primarily managed with chemical seed coatings containing fungicides and oomicides. However, improved knowledge on spermosphere microbiology and ecology would support the successful inclusion of alternative strategies to chemical seed treatments. For example, biocontrol of *Pythium* from seed-applied Enterobacter cloacae could be achieved by metabolizing long-chain fatty acids, which otherwise stimulated the germination of *Pythium* sporangium (5, 12, 31). Studies on the spermosphere either have been culture based or, more recently, have focused on the contribution of the indigenous seed microbiome by using sterile or soilless growth conditions or preimbibed or pregerminated seeds, which do not sample the spatial and temporal properties of the spermosphere soil (18, 32–34). Indeed, natural seed-associated epiphytes and endophytes compete with pathogens (35, 36). While commendable, these studies have largely ignored the influence of the initial seed exudate release on the spermosphere soil microbes. Therefore, a mechanistic understanding of the complex interactions in spermosphere soil will aid in novel treatments for seed and seedling pathogens and help our understanding of plant microbiome assembly.

However, one major challenge in studying spermosphere soil using high-throughput culture-independent techniques may be a lack of a quick and efficient method of collecting spermosphere soil (3). In this study, we aimed to capture changes in microbial diversity in the spermosphere soils as soybean and cotton seeds underwent phases I and II of seed germination (i.e., pre-radical emergence). We sampled the spermosphere soil of cotton and soybean by constraining the soil zone within wells of a 12-well plate and sampling precisely 3 to 6 mm of soil around an imbibing seed with an appropriately sized cork borer, extracting DNA, and sequencing the 16S and internal transcribed spacer (ITS) ribosomal DNA (rDNA) from cotton and soybean spermosphere soil. We hypothesized that seeds would imbibe water rapidly and follow previously established phases of exudate release, which would alter microbial diversity and co-occurrence patterns. We also hypothesized that spermosphere soil microbial diversity would be distinct based on crop species. Therefore, the objectives of this study were 2-fold: (i) to characterize the bacterial and fungal microbial communities associated with cotton and soybean spermosphere soil compared to control soil and (ii) to determine how microbial diversity and co-occurrences change over time as a seed imbibes water.

## RESULTS

### Sequencing outputs.

Mock operational taxonomic units (OTUs) for the fungi and prokaryotes made up 99.9% of the composition of the positive controls, indicating minimal cross-contamination. Nine prokaryotic OTUs were filtered after detection in negative-control samples, resulting in 2,090,814 16S V4 reads of 8,088 OTUs across 71 samples with a median read depth of 29,237 reads per sample. Nineteen fungal OTUs were detected in negative controls and taken out, resulting in 2,534,301 ITS1 reads with 1,904 fungal OTUs across 69 samples and a median read depth of 37,933 reads per sample. Rarefaction curves indicated that much of the diversity was adequately captured (see Fig. S2 in the supplemental material).

### Prokaryote community dominance correlates with water imbibition.

**FIG 1:**
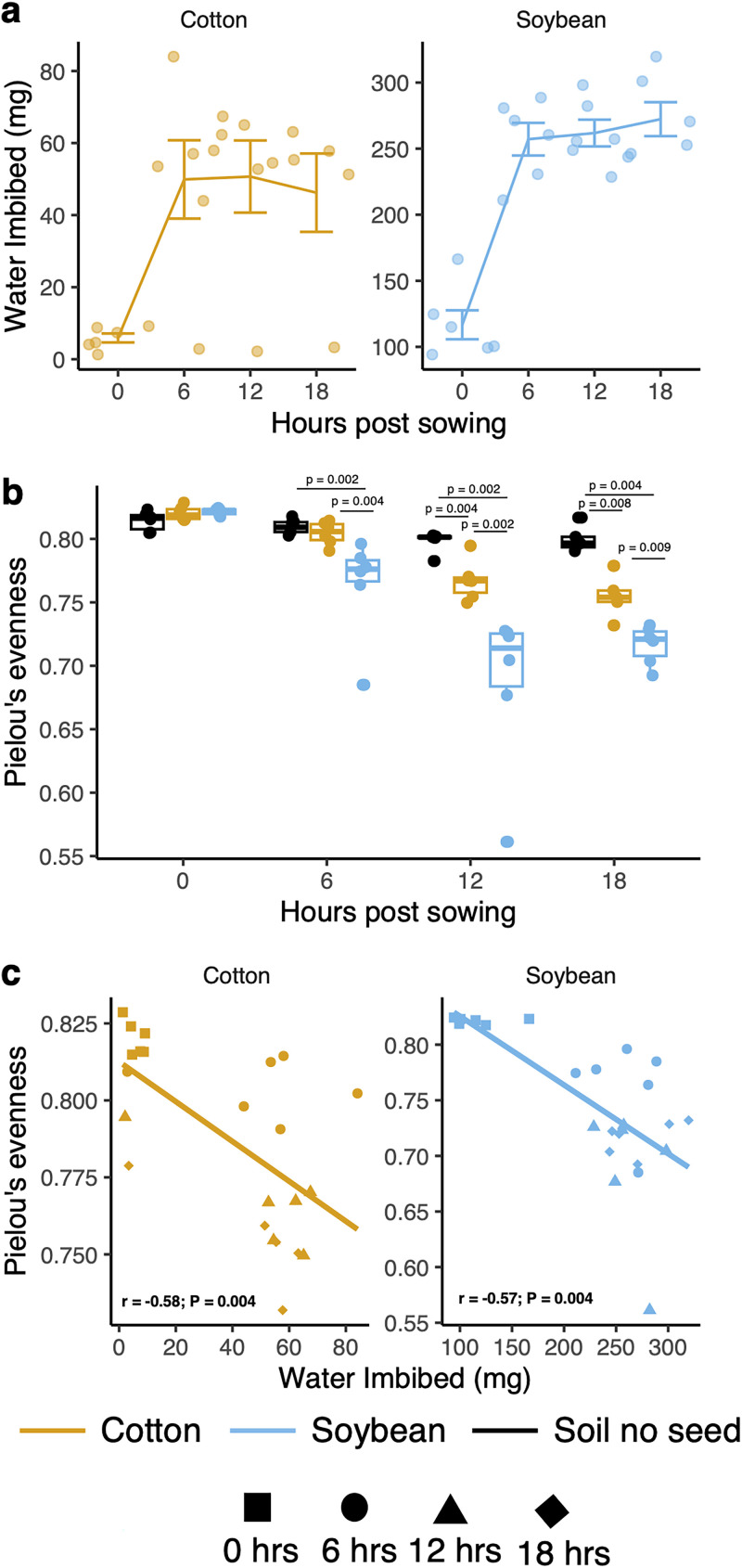
Prokaryote spermosphere evenness follows water imbibition. (a) Water imbibition over time for cotton and soybean seeds (*n* = 6). (b) Prokaryote evenness over time in control soil, soybean spermosphere soil, or cotton spermosphere soil. Soybean had significantly lower evenness (higher dominance) after 6, 12, and 18 h than did control soil. Cotton followed the same trend but was significantly less even after 12 and 18 h. Comparisons were made with Wilcox signed-rank test (α = 0.05; *n* = 6). (c) Spearman correlation between prokaryote evenness and water imbibition.

The individual measurement of seed weight for soybean and cotton seeds before and after spermosphere collection indicate that water was imbibed from the surrounding soil (Fig. 1a). Overall, soybean seeds imbibed more (250 to 300 mg) than cotton seeds (50 to 80 mg) and both seeds increased in seed weight within the first 6 h, indicating imbibition within this time frame, and then plateaued after 6 h.

We observed a reduction in prokaryote evenness (or increase in dominance) over time in spermosphere soils but not in control soil (Fig. 1b). At time zero, there was no significant difference in the evenness of prokaryote taxa (*P = *0.18), as expected. At 6 h, we observed a reduction in the evenness of prokaryote taxa in soybean spermosphere soils compared to cotton spermosphere (*P = *0.004) or control (*P = *0.002) soil. At 12 and 18 h, both cotton and soybean spermosphere samples had significantly reduced evenness compared to control soil and each other (*P ≤ *0.009). Prokaryote evenness was significantly negatively correlated with water imbibition, meaning that as seeds imbibed water and released exudates, prokaryote communities became more dominant (for cotton, *r* = −0.58 and *P < *0.001; for soybean, *r* = −0.57 and *P = *0.004) (Fig. 1c). However, the crop did not alter prokaryote richness or phylogenetic diversity compared to that of control soil. Prokaryote richness and phylogenetic diversity dropped significantly over time regardless of habitat (*P < *0.001). Additionally, there was no consistent evidence that fungal richness or evenness was altered in spermosphere samples compared to that of control soil. Still, a few samples of soybean spermosphere soil and control soil dropped in evenness after 18 h (Fig. S3), becoming more dominant in fungal OTU 2 (fOTU2) Fusarium (Fig. S4).

### Spermosphere prokaryote composition depends on the crop.

**FIG 2:**
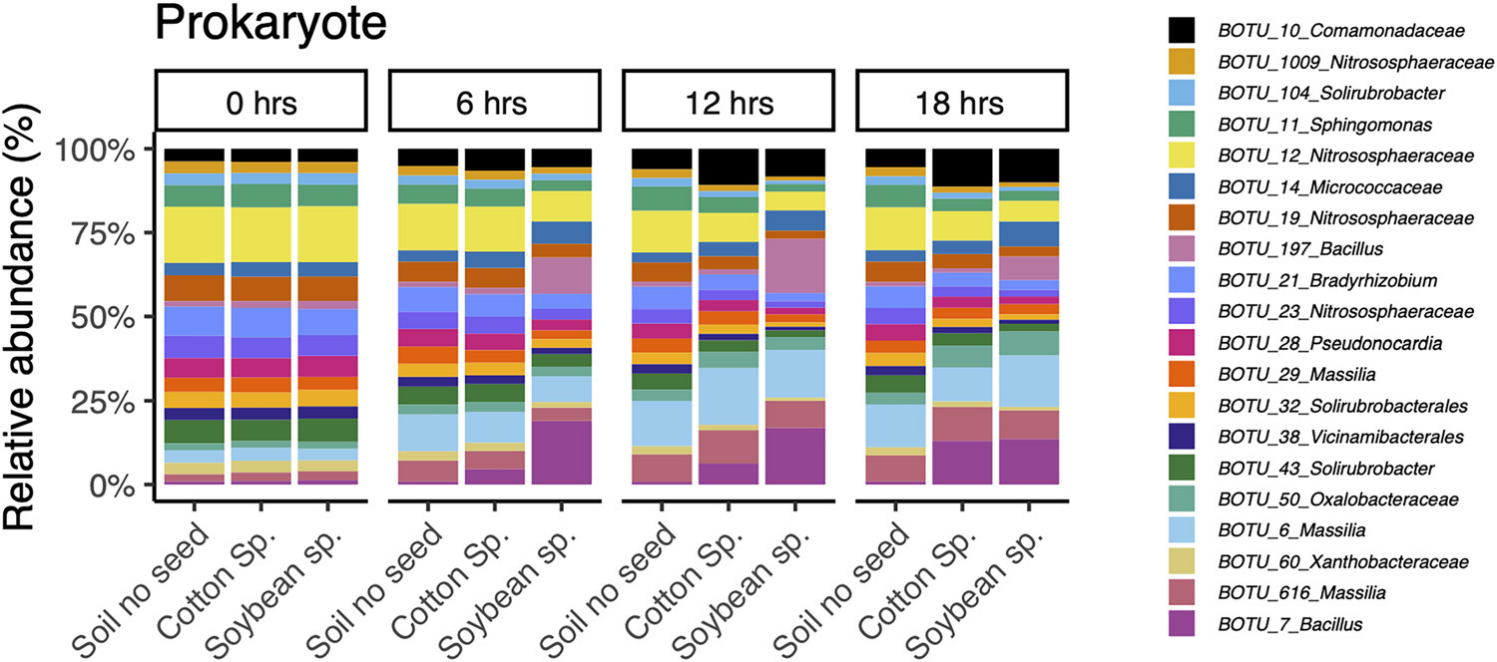
Composition of the most abundant prokaryotic OTUs changes over time. Relative abundance of the top 20 most abundant prokaryote OTUs shifts over time within soybean spermosphere soil, cotton spermosphere soil, or control soil.

**FIG 3:**
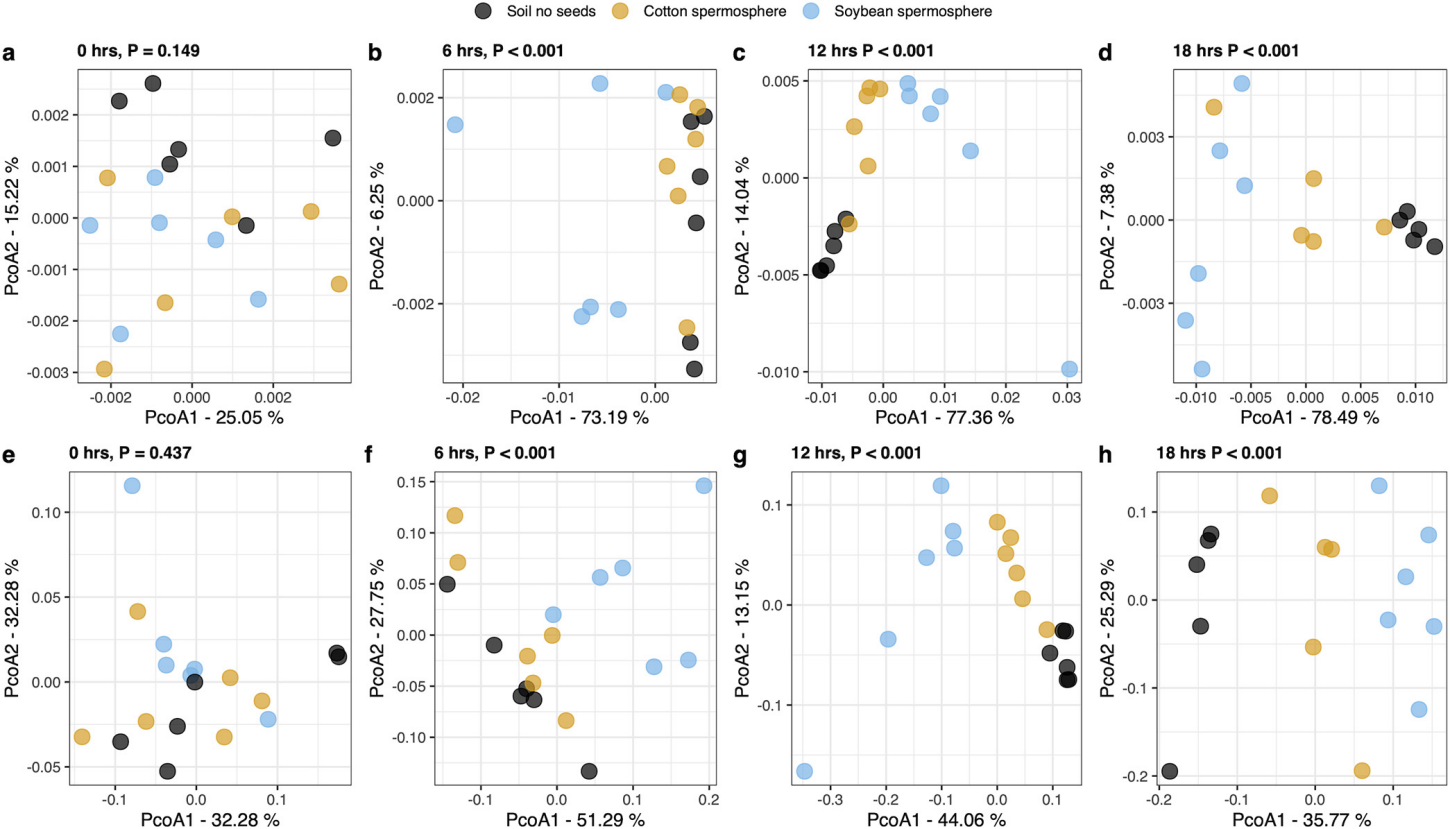
Prokaryote spermosphere composition changes over time and crop. (a to d) Principal-coordinate analysis based on weighted UniFrac distances. (e to h) Principal-coordinate analysis based on Bray-Curtis distances. Reported significance values above each plot are the results of a permutational test of variance (α = 0.05; *n* = 6). Accompanying dispersion tests are shown in Table 1.

Spermosphere soils had different prokaryote community compositions than control soil. A visualization of the change in the most abundant prokaryote composition over time is shown in Fig. 2. Prokaryote communities were driven by habitat (*P < *0.001), time since sowing (*P* < 0.001), and interaction of these two factors (*P* < 0.001) (Table S1). The interaction prompted a closer look into the differences observed between crops by splitting the data by time point (Fig. 3; Table 1). At 0 h, no significant difference in prokaryote communities existed between bulk soil and soybean and cotton spermospheres, as expected (Bray-Curtis, *P = *0.452; weighted UniFrac, *P = *0.192). However, as early as 6 h, we observed significant differences between control soil and spermosphere soil samples (Bray-Curtis, *P < *0.001; weighted UniFrac, *P < *0.001). Differences were extended through 12 h (Bray-Curtis, *P < *0.001; weighted UniFrac, *P < *0.001) and 18 h (Bray-Curtis, *P < *0.001; weighted UniFrac, *P < *0.001), when it was clear that the spermosphere formed unique community compositions within soybean or cotton. Further, differences in multivariate dispersions were not observed, supporting true differences in centroids rather than group dispersions (Table 1). This trend was not observed with fungi. Time did alter fungal community composition (*P = *0.01), but there was no evidence that soybean or cotton altered fungal community composition compared to that of control soils (*P = *0.09).

**TABLE 1:** PERMANOVA and dispersion for prokaryote communities separated by time post sowing based on Bray-Curtis and weighted UniFrac distances^*a*^

Dissimilarity metric	Hours post sowing	PERMANOVA			Dispersion	
		*R* ^2^	*F*	*P*	*F*	*P*
Bray-Curtis	0	0.113	0.959	0.437	0.937	0.407
	6	0.273	2.815	<0.001	0.539	0.606
	12	0.424	5.517	<0.001	0.828	0.533
	18	0.417	4.648	<0.001	0.092	0.911
Weighted UniFrac	0	0.142	1.238	0.192	1.898	0.190
	6	0.491	7.245	<0.001	2.538	0.066
	12	0.609	11.675	<0.001	1.186	0.376
	18	0.696	14.863	<0.001	1.401	0.304

a Permutational analysis of variance (PERMANOVA) and dispersion tests were performed on 999 permutations. The *df* was 2 in all cases.

### Enriched prokaryotes in the spermosphere have unique and shared taxa among crops.

**FIG 4:**
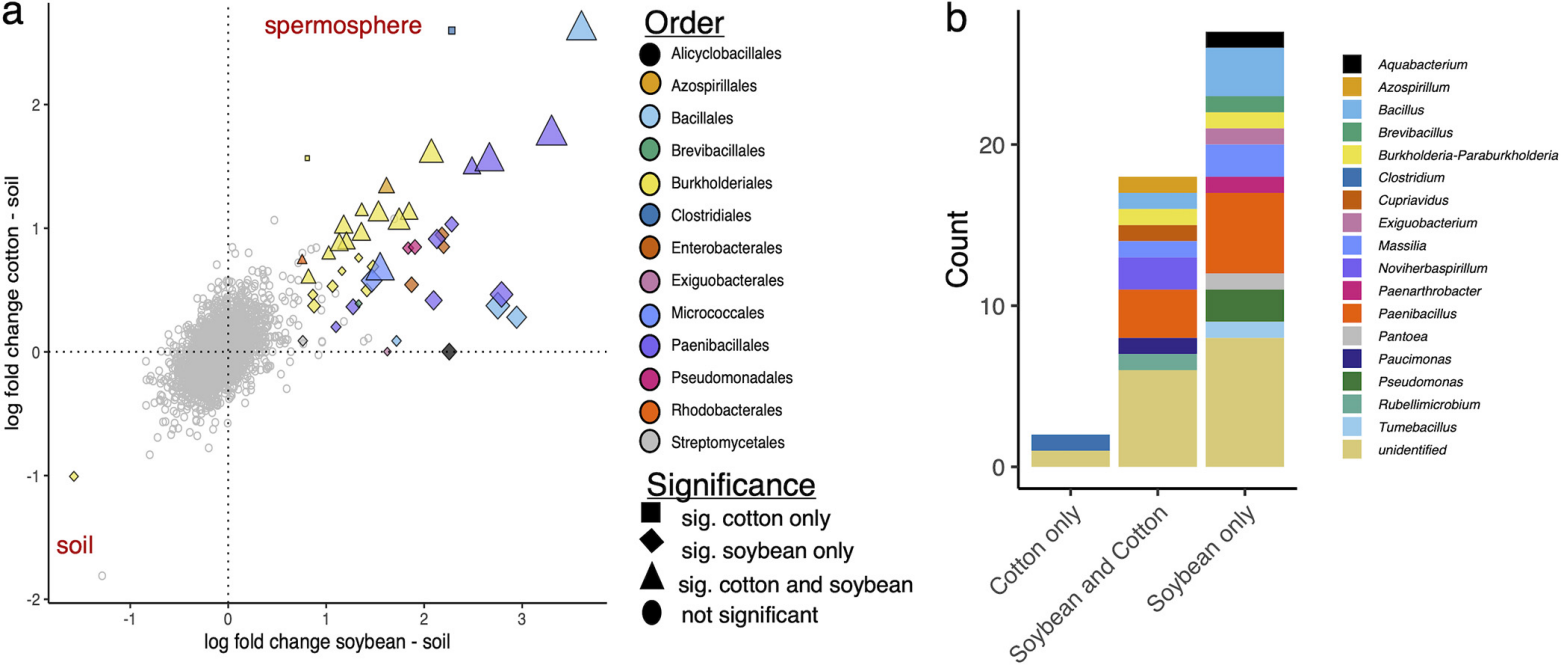
Differential abundance of prokaryote OTUs shows enrichment of specific taxa within the spermosphere. (a) Points represent individual OTUs. Positive values on the *x* axis indicate that the OTU was numerically more abundant in a soybean spermosphere than in soil without a soybean seed. Similarly, positive *y* axis values indicate that the OTU was numerically more abundant in a cotton spermosphere than in soil without a cotton seed. Colored points are pOTUs detected as significantly different in soybean or cotton. Gray circle points are nonsignificant. Point shape indicates significance in one crop or both. Points are colored by the prokaryote order. Significance was determined within the ANCOM-BC2 algorithm with a Holm-Bonferroni correction (α = 0.05). (b) Composition of significantly enriched pOTUs colored by genus.

Differential abundance analysis determined sets of prokaryotic OTUs (pOTUs) significantly enriched in the spermosphere of cotton and soybean compared to that of control soil (Fig. 4a; Table S2). Ninety-four percent of the enriched taxa belonged to *Proteobacteria* (57% [*n* = 27]) and *Firmicutes* (36% [*n* = 17]). The remaining three belonged to *Actinobacteria*. Within the *Proteobacteria*, the enriched taxa were spread across 10 prokaryote families, with the most enriched taxa in the *Oxalobacteraceae* (41% [*n* = 11]). The majority of the enriched *Proteobacteria* were unidentified at the genus level (*n* = 12) but included *Massilia* (*n* = 3), *Noviherbaspirillum* (*n* = 2), *Burkholderia*/*Paraburkholderia* (*n* = 2), *Aquabacterium* (*n* = 1), Pseudomonas (*n* = 2), *Cupriavidus* (*n* = 1), *Pantoea* (*n* = 1), *Paucimonas* (*n* = 1), *Rubellimicrobium* (*n* = 1), and *Azospirillum* (*n* = 1) (Fig. 4b). Within the *Firmicutes*, all but one pOTU belonged to the *Bacilli* class with the genera *Paenibacillus* (*n* = 8), *Bacillus* (*n* = 4), *Brevibacillus* (*n* = 1), *Exiguobacterium* (*n* = 1), and *Tumebacillus* (*n* = 1). Many enriched taxa were shared between cotton and soybean (*n* = 18), indicating that similar taxa take advantage of release of exudates from seeds (Fig. 4b). All the enriched pOTUs were present in control soil samples, meaning that it was unlikely that they originated from the seed but were present in the soil and proliferated upon exudate release from the seeds.

### Cotton and soybean spermosphere networks are more connected and have distinct microbial hub taxa.

**FIG 5:**
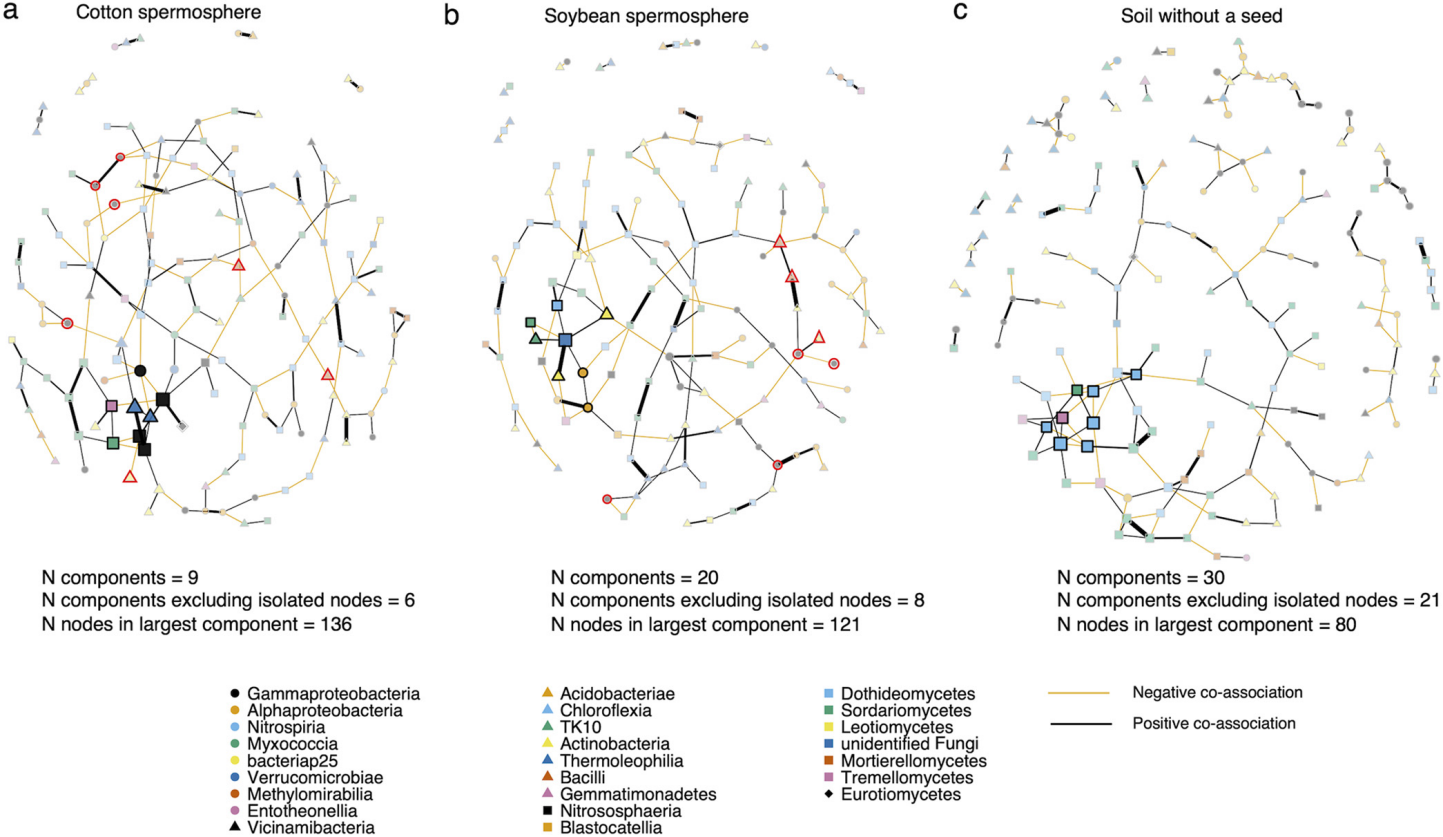
Spermosphere soils have different topological properties and different sets of hub taxa than control soil. Networks for cotton spermosphere soil (a), soybean spermosphere soil (b), and control soil (c) were constructed with the same set of taxa. Nodes with different shape and colors indicate the prokaryote or fungal class. Less transparent nodes are significant hubs based on eigenvector centrality above the 95% quantile. Nodes with red outlines were significantly enriched in a spermosphere as detected in Fig. 3.

Cotton and soybean spermosphere networks were compared to each other and to the control soil to determine if they contained different topologies or different sets of network hubs and to determine the centrality of spermosphere-enriched taxa. Overall, network topology parameters were similar between networks except for the number of separate components. In other words, the spermosphere soil networks consisted of fewer subnetworks than the control soil network (Fig. 5). For example, the control soil network contained 30 components and 80 nodes within the largest component. Soybean and cotton spermosphere soil networks had more nodes within the largest component (cotton, 136, and soybean, 121). However, control soil and a slightly higher positive edge percentage (61% control soil, 58% cotton spermosphere soil, 52% soybean spermosphere soil) (Table S3).

Comparison between central nodes was significantly different, indicating that the hub taxa were different between networks given the same sets of taxa used to construct the networks (Table 2). Hub taxa for cotton consisted of six prokaryotic OTUs and two fungal OTUs. Cotton prokaryote hubs consisted of three *Archaea* OTUs in the *Nitrososphaeraceae* family (pOTU1009, pOTU19, and pOTU12), two OTUs from the *Gaiellales* (pOTU46 and pOTU119), and pOTU29 *Massilia* (Fig. 5a; Table 3). Fungal cotton hubs were fOTU56 Fusarium chlamydosporum and yeast fOTU36 Hannaella oryzae (Fig. 5a; Table 3), whereas the soybean network had three fungal hub taxa: fOTU64 *Helicoma*, fOTU10 Bartalinia pondoensis, and an unidentified fungus in fOTU36 (Fig. 5b; Table 3). Prokaryote hub taxa in the soybean spermosphere network contained pOTU11 *Sphingomonas*, pOTU132 *Nocardioides*, pOTU1559 *Chloroflexi* TK10, pOTU36 *Angustibacter*, and pOTU349 *Methylobacterium*/*Methylorubrum* (Fig. 5b; Table 3). The network from the control soil contained only fungal hubs, different than the identities of spermosphere fungal hubs except for fOTU36 *Hannaella oryzae*. Spermosphere-enriched taxa included in the network analysis were not hub taxa, indicating that although enriched in a spermosphere, other microbial taxa besides the enriched taxa may play an essential role in maintaining spermosphere network structure (Fig. 5).

**TABLE 2:** Comparison of network centrality parameters

Centrality network parameter	Cotton vs soybean		Cotton vs control soil		Soybean vs control soil	
	Jaccard index^*a*^	*P*	Jaccard index	*P*	Jaccard index	*P*
Degree	0.034	<0.001	0.128	0.001	0.044	<0.001
Betweenness centrality	0.154	<0.001	0.156	0.001	0.154	<0.001
Closeness centrality	0.152	<0.001	0.246	0.092	0.206	0.019
Eigenvector centrality	0.169	0.003	0.188	0.007	0.206	0.019
Hub taxa	0.000	0.002	0.067	0.019	0.000	0.001

a A Jaccard index of 1 indicates perfect agreement between soybean or cotton spermosphere networks.

**TABLE 3:** Hub taxa identified for each habitat

Soil	OTU	Taxonomy
Cotton spermosphere soil	pOTU46	*Gaiellales*
	pOTU119	*Gaiellales*
	pOTU29	*Massilia*
	pOTU1009	*Nitrososphaeraceae*
	pOTU19	*Nitrososphaeraceae*
	pOTU12	*Nitrososphaeraceae*
	fOTU36	*Hannaella oryzae*
	fOTU56	Fusarium chlamydosporum
Soybean spermosphere soil	pOTU1559	*Chloroflexi* TK10
	pOTU132	*Nocardioides*
	pOTU36	*Angustibacter*
	pOTU11	*Sphingomonas*
	pOTU349	*Methylobacterium*/*Methylorubrum*
	fOTU64	*Helicoma*
	fOTU36	*Fungi*
	fOTU10	*Bartalinia pondoensis*
Soil without a seed	fOTU1	Stagonosporopsis oculi-hominis
	fOTU13	*Teichosporaceae*
	fOTU15	*Cucurbitariaceae*
	fOTU17	Alternaria tenuissima
	fOTU22	*Neopestalotiopsis*
	fOTU32	*Pleosporales*
	fOTU36	*Hannaella oryzae*
	fOTU4	Cladosporium cladosporioides

## DISCUSSION

To our knowledge, this is the first study to use culture-independent sequencing to study soybean and cotton spermosphere soil microbiomes during the first phases of seed germination. The advancement that allowed this was the method that constrained nonsterile soil to wells within a 12-well plate and sampled around an imbibing seed with a cork borer. The technique enabled the precise and efficient collection of spermosphere soils as defined in space and time (1), which we believe represents a more realistic spermosphere habitat. The focus on spermosphere soil in the first phases of seed germination differs from other studies that preimbibe or pregerminate seeds under axenic conditions. We hypothesized and observed a rapid increase in water imbibition followed by a plateau characterizing phase I and phase II of seed germination. Prokaryote community structure changed in as little as 6 h for soybean and 12 h for cotton. We did observe that crops had unique prokaryote community structures in the spermosphere that were distinct from those in the control soil, typified by differences in network hub taxa and network topologies. The differing hub taxa demonstrate that other taxa besides the enriched taxa are integral to each crop’s spermosphere community structure. However, despite the differences in composition and hub taxa, among the most important observations was the commonality in the enriched copiotrophic taxa with a long history of benefiting plant growth, such as *Bacillus*, *Paenibacillus*, *Burkholderia*, *Massilia*, *Azospirillum*, and Pseudomonas.

In this study, we further defined the development of the spermosphere of cotton and soybean at 6 to 12 h after sowing, which aligns with previous studies of increased spore germination and full colonization of cotton seeds by Pythium ultimum 12 h after sowing (10, 11). We observed an increase in water imbibed by both cotton and soybean seeds in the first 6 h, which is consistent with previous reports that documented increased water imbibition and exudation within the first few hours after sowing (15). Imbibition ceased representing phase II of germination, indicating the saturation of nutrient reserves and synthesis of products required for the extending radicle (1).

Similar to the case with several other studies (37–39), we observed that the soil microbes responded to seed exudates and dominated the spermosphere microbiomes. We observed changes in phylogenetic dissimilarity between crops, and since phylogenetically similar species are more likely to share ecological characteristics and functional traits (40), it may be expected that the spermosphere communities in our study changed in a functional capacity (37). However, we observed various spermosphere prokaryote compositions in different plant species, which may highlight the importance of sample collection at the initial stages of seed germination and imbibition rather than at later hours potentially after radicle emergence. Additionally, future studies should also include other soils with inherently different communities to understand the contributions of different soil microbial pools to formation of the spermosphere.

The difference between crops may have also been due to differences in the amount of water imbibed. We noticed that soybean imbibed more than cotton seeds, likely due to seed size (41, 42). Different varieties of common bean have been shown to differ in the amount of seed exudates, with larger seeded varieties releasing more exudates (43). Thus, we speculate that the greater and faster turnover in microbial communities of the soybean spermosphere than for cotton may be due to the larger size of soybean seeds and increased exudation, which potentially supported a larger habitat for the microbes to occupy. It also leaves the question of whether microbial communities would have converged on similar compositions if a later sampling point had been included.

Regardless, as a result of water imbibition and seed exudation, we observed a change in dominance in the spermosphere microbiome over time with both crops. Upon revealing enriched taxa in soybean and cotton spermosphere soils, we found some commonalities. Importantly, *Bacilli* were enriched in the spermosphere soils of both crops. Since these *Bacilli*, including *Tumebacillus*, *Paenibacillus*, and *Bacillus*, have historically been associated with plant growth promotion and disease protection and have commercial potential, they were notable. Our finding indicates their ability to utilize seed exudates quickly for growth. Seed exudates have been reported to induce chemotaxis, seed colonization, and biofilm formation of Bacillus amyloliquefaciens (Bacillus velezensis) by enhancing active cell division (44). Paenibacillus polymyxa isolated from wheat and peanut rhizosphere increased the survival of Arabidopsis thaliana in the presence of the oomycete pathogen Pythium aphanidermatum when applied as root treatment (45). Identifying these enriched taxa is important for prioritizing future work on a mechanistic understanding of the spermosphere microbial interactions that will improve the development of efficacious biologically based disease solutions (1, 46).

In terms of seed versus soil origin, there were OTUs with a low relative abundance and low occupancy that occurred only in cotton or soybean spermosphere samples and were absent from the soil. However, we hesitate to conclude that they originated from the seed without directly identifying seed epiphytes and endophytes since it was impossible to know if the unique microbes were seed associates colonizing the spermosphere or if they were rare members of the soil that were only present in spermosphere samples by chance. Furthermore, surface-disinfecting seeds used in this study likely reduced the number of seed epiphytes that would colonize the spermosphere. The implications of surface-disinfecting seeds have been argued elsewhere (3, 4, 34). Another limitation of our approach that limited our ability to identify seed-associated microbes may be the use of OTUs rather than amplicon sequence variants (ASVs) or zero-radius OTUs (zOTUs). A finer clustering method may be better suited to studying the transmission of seed-associated microbes into the spermosphere since genotypes originating from the seed may be different than genotypes originating from the soil whereas using 97% OTUs may cluster both genotypes into the same OTU. We recognize that microbes originating from the seed can colonize seedlings and other plant organs, which can alter plant health (4, 16–19, 32, 47–49). For example, it was recently demonstrated that crop flowers sprayed with a beneficial bacterium can colonize endosperm and transmit to germinating seeds (49). While the importance of seed-associated microbes on plant health is evident, little is known about seed endophytes and interactions with horizontally acquired soil organisms, which tend to contribute a large portion of the microbial diversity to the seedling microbiome (2, 18, 37).

In terms of the microbial networks, we observed different hubs and different topologies given the same set of taxa used for network construction. While fungal diversity was not altered in this study, fungal OTUs were identified as hubs, potentially demonstrating meaningful interactions within spermosphere soil. Of most interest was the yeast *Hannaella* since this organism is commonly found in soils, the phyllosphere, and as part of the core seed and phyllosphere microbiome (19, 50, 51). *Dioszegia*, in the same family as *Hannaella*, was identified as a network hub in the phyllosphere (52), and the closely related yeast *Bullera* has been a network hub of the soybean phyllosphere (53). These yeasts are generally nonpathogenic, but their ecological role is poorly understood (54). The prokaryote hubs were also intriguing because cotton contained several *Nitrososphaeraceae* pOTUs, which likely are involved with ammonia oxidization in soils (55). Cotton spermosphere hubs also had a *Massilia* pOTU. *Massilia* is known for below-ground associations and the ability to solubilize phosphate (56), but it has also been found as a hub in above-ground plant tissues (53). For soybean, *Sphingomonas* and *Methylobacterium*/*Methylorubrum* pOTUs were notable network hubs since these genera have been demonstrated to be abundant in the phyllosphere and core seed microbiome and produce plant growth-promoting hormones and UVA-absorbing compounds (57, 58). The difference in hub taxa between crops demonstrates that soybean and cotton construct unique microbial communities early in life, which may have plant health consequences at or beyond the spermosphere stage.

However, spermosphere-enriched pOTUs were not identified as network hubs; instead, they were located more peripherally in the networks, indicating that they may be copiotrophs responding quickly to the availability of carbon-rich exudates from the seeds (59). Spermosphere networks were more connected with larger components than the soil network. Increased soil network complexity was associated with increased microbiome function (60). Therefore, it may be hypothesized that seed exudates help stimulate associations between organisms or subcommunities and form more connected or stable communities. However, further research is needed to determine how topological features of networks are associated with plant health and why hub taxa connect to other taxa and help assemble plant microbiomes.

The technique used in this study enabled quick and efficient collection of spermosphere soil within phases I and II of seed germination and showed the enrichment of beneficial copiotrophic taxa. However, these copiotrophic taxa were not central to microbial networks. This technique could easily be applied to other sequencing methods like metagenomics or metatranscriptomics for a better understanding of spermosphere soil microbiome functions. Coupled with sequencing, the seed microbiomes will be powerful to study interactions between seed and seedling pathogens, chemical or biological seed treatments, and interactions with pathogens in the spermosphere, thereby improving knowledge of spermosphere ecology, which will lead to improved understanding of the plant microbiome.

## MATERIALS AND METHODS

### Soil collection and preparation.

The soil used in this study was collected from a field used for cotton, soybean, and corn rotation from Prattville Agricultural Research Unit in Prattville, AL (32.42533, −86.4452), since this soil showed consistent emergence of both cotton and soybean in preliminary experiments (data not shown) and was not known to contain a high abundance of any specific seedling pathogen. Approximately 3 L of soil from the top 10 cm was collected and transported to the lab. The soil was sieved to eliminate stones and pebbles and air dried for 24 h to ensure homogeneity in water content. The soil was used immediately after air drying. Six to seven grams (6 mL) of soil was transferred to each well of the 12-well microtiter plates (VWR American catalog no. 10861-556), containing three 1-mm holes in the bottoms of all wells for drainage. Each well in the 12-well microtiter plates measured a total volume of 6.8 mL, and the depth of each well of a 12-well plate was 15 mm, with a width of 23 mm. Each well containing soil was watered with 1.5 mL of sterile water (25% soil moisture), and the water was allowed to circulate for 1 h before the seeds were sown.

Nontreated Williams-82 soybean and nontreated delinted Delta Pine 1646 B2XF cotton were used in this study and were sorted to discard discolored seeds or seeds with cracked seed coats (11). The weight of individual dry seeds was recorded before use and after imbibition to record how much water was imbibed. The initial weight of soybean seeds was between 170 and 250 mg, and cotton seeds weighed between 60 and 110 mg. The average size of soybeans used was between 5 and 8 mm in diameter, and the soybeans were spherical. Cotton seeds were more oblong, 3 to 4 mm in diameter and 10 millimeters long. Seeds were surface sterilized by soaking in 6% bleach solution for 10 min in a sterile petri dish and washed three times with sterile distilled water. Seeds were surface sterilized to maximize the effect of seed exudates on the growth of microbes from the soil. Six replicate seeds were sown into the center of individual wells, halfway into the 15-mm depth of the well, using flamed forceps. Wells containing only soil without a cotton or soybean seed were used as a control. The 12-well microtiter plates were placed in a planting tray covered with a lid to keep the soil from drying. Planting trays containing 12-well microtiter plates were placed inside a growth chamber at 25°C.

### Collection of spermosphere.

**FIG 6:**
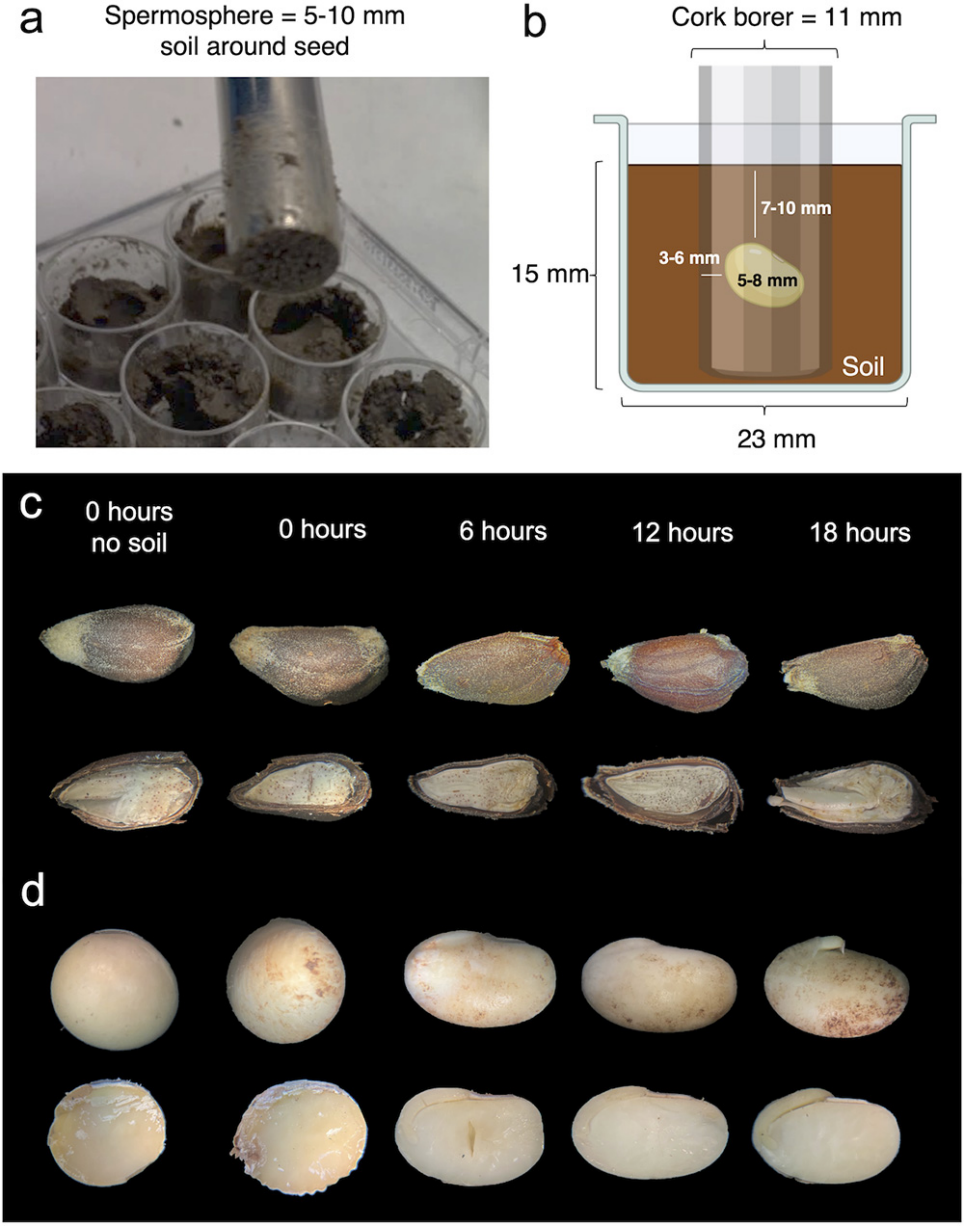
Technique used for sampling spermosphere soils around individual seeds and the resulting seed morphology over time. The spermosphere is defined as the 5 to 10 mm of soil directly surrounding a seed. (a) Photo demonstrating the sampling technique of spermosphere soil contained within an 11-mm cork borer. (b) Sampling with an 11-mm cork borer inside the confining space within wells of a 12-well plate allowed direct and controlled sampling of the spermosphere around single seeds. (c and d) Cotton (c) or soybean (d) seed embryonic tissue was not visibly hydrated after 0 h. However, after 6 h seed coats softened and embryonic tissue was hydrated, indicating that phase I of seed germination (i.e., seed hydration) had occurred. At 18 but not at 12 h both crops had visibly emerged radicles, indicating the end of phase II germination between 12 and 18 h.

Spermosphere soil samples were collected at 0, 6, 12, and 18 h after sowing. Wells containing only soil were sampled as a control and are here referred to as control soil. Spermosphere soil and control soil samples were collected using an 11-mm cork borer cleaned of soil with 70% ethanol and flame sterilized between samples. The 11-mm cork borer was specifically used since the spermosphere is defined as the first 5 to 10 mm of soil around a germinating seed (1) and allowed soil collection within this range based on the seed sizes stated previously. Therefore, given the size of the well, the volume of soil used, and the seed sizes, the spermosphere soil sampled consisted of 3 to 6 mm on either side of a soybean seed and 7 to 10 mm above and below a soybean. Similarly, the spermosphere soil sampled for cotton consisted of 7 to 8 mm on either side and 5 to 10 mm above and below the seed. The sampling procedure for soybean is shown in Fig. 6a and b, and seed morphology at each time point sampled is shown in Fig. 6c and d. After 6 h we observed initial seed hydration resulting in softening of the seed coat and embryotic tissue within 6 h (phase I). After phase I, phase II was complete once the radicle broke through seed coats by 18 h (Fig. 6c and d). In preliminary experiments, bacterial populations in spermosphere soils sampled with this method increased significantly by 1.15 log in soybean and about 0.8 log in cotton compared to those in control soil (see Fig. S1 in the supplemental material).

Spermosphere soil containing the seed inside the core within the cork borer was transferred into sterile envelopes, and 0.25 mL was immediately transferred to 2-mL disruptor tubes (E.Z.N.A. soil DNA; Omega Bio-tek, Norcross, GA) and then stored at −80°C until DNA extraction. The remaining soil clinging to the seed was washed off, the seed was blotted dry of excess water, and the weight of the seed was recorded after sample collection and compared to the initial individual seed weight to determine the water imbibed by each seed.

### DNA extraction, amplification, and sequencing.

The total DNA was extracted from the spermosphere and control soils following the manufacturer’s recommendation. Amplification and library construction of 16S or ITS ribosomal DNA (rDNA) was performed with a three-step PCR (53, 61). Briefly, the 16S region of the rDNA was amplified using the forward and reverse primers 515F and 806R. Amplification of the ITS used the primers ITS1F and ITS4. Following the amplification of the respective rDNA regions, the amplicons were linked to variants of frameshift primers, and then a 10-bp barcode was added for sample identification. Library negative controls consisted of DNA extraction without soil and no-template PCR water controls. The ZymoBIOMICS microbial community DNA standard (Zymo Research, Irvine, CA) was used as a positive-control mock community. A fungal synthetic mock community was used as a positive control for fungi (62). DNA amplification was confirmed with gel electrophoresis, and successfully amplified libraries were normalized using a SequalPrep normalization plate kit (Thermo Fisher, USA). Normalized amplicons were then pooled and concentrated 20:1 using 50K Dalton Millipore filters (Sigma-Aldrich, USA). The pooled library was cleaned using AMPure XP beads at a ratio of 0.7× (Beckman Coulter, USA). Cleaned amplicon pools were verified by gel electrophoresis, quantified using a Qubit fluorometer (Thermo Fisher, USA), and sequenced on an Illumina MiSeq 2 × 300-bp platform using the v3 600 cycles kit at SeqCenter LLC (Pittsburgh, PA). Primers and cycling parameters to construct libraries were the same as described by Noel et al. (50).

### Read processing.

The quality of demultiplexed reads was assessed using FastQC, and primer sequences were removed using cutadapt 4.0 (63). Prokaryote 16S V4 sequences were merged using VSEARCH 2.21.1 (64). Only forward fungal ITS1 reads were used since reverse reads did not overlap. Fungal reads were trimmed to remove the conserved 18S regions. Reads were then truncated to equal length (fungi, 200 bp; prokaryotes, 300 bp) and quality filtered using VSEARCH 2.21.1 with an expected error threshold of 1.0. Singletons were removed and reads *de novo* clustered based on 97% identity into prokaryotic OTUs (pOTUs) or fungal OTUs (fOTUs) using USEARCH v11.0.667, which includes a chimera detection and removal step (65, 66). The resulting pOTUs were aligned using MAFFT v7.505 (67), and a phylogenetic tree was estimated using FastTree v2.1.20 (68) and then midpoint rooted with FigTree v1.4.4 (69). Taxonomy was assigned to resulting pOTUs using the SINTAX algorithm (70) against the SILVA 138.1 database (71). Fungal taxonomy was assigned using the Ribosomal Database Project’s naive Bayesian classifier algorithm against the UNITE fungal ITS database version 9.0 (72).

### Data analysis.

Data were primarily analyzed using phyloseq v.1.34.0 (73) and vegan v2.5-7 (74) of the statistical software R v.4.2.2. All plots were generated using the data visualization package ggplot2 v.3.3.5 (75). Contaminant OTUs detected in the negative controls were removed with decontam v1.10.0 (76). Prokaryote samples with fewer than 10,000 reads were discarded. Fungal samples with fewer than 1,000 reads were discarded due to low sequencing coverage.

Richness, Pielou’s evenness (77), and Faith’s phylogenetic diversity (78) were used to determine within-sample diversity differences using Kruskal-Wallis one-way analysis of variance. Read counts were then normalized using cumulative sum scaling with metagenomeSeq v1.32.0 (79) and subjected to principal-coordinate analysis based on Bray-Curtis distances for fungi and prokaryotes or weighted UniFrac distances for prokaryotes only. This analysis was followed by a permutational analysis of variance (PERMANOVA) implemented with the adonis2 function to determine the differences in centroids of the prokaryote or fungal communities across time points and soil versus spermosphere. Differences in multivariate dispersion were also evaluated using the betadisper function.

Differential abundance analysis was conducted with Analysis of Compositions of Microbiomes with Bias Correction version 2 (ANCOM-BC2) (80). Significantly different OTUs were detected based on Holm-Bonferroni-corrected *P* values. Then, microbial co-occurrence networks with prokaryotes and fungi were constructed using SpiecEasi v1.1.2 (81) and compared between soybean spermosphere soil, cotton spermosphere soil, and control soil using NetCoMi v1.1.0 (82). For network construction, spermosphere and control soil samples at 12 and 18 h were filtered to a common set of taxa with a relative abundance above 0.001% and occupancy above 90%. Co-occurrence association matrices were estimated using the Meinshausen and Bühlmann algorithm with the “nlambda” set to 100, sampled 100 times, and with the “lambda.min.ratio” set to 10^−1^. All resulting networks contained stability values of 0.048 or above, close to the 0.05 StARS algorithm stability target. Association matrices for spermosphere soils or control soil were compared using the netAnalyse function from NetCoMi. Hub taxa were identified based on eigenvector centrality values above the 95% quantile of a fitted log-normal distribution. Comparison of the hub taxa composition was based on the Jaccard similarity index.

### Data availability.

The data files and scripts used for this analysis are available on GitHub (https://github.com/Noel-Lab-Auburn/SpermosphereMicrobiome2022) (83). Raw sequence reads were deposited to the sequence read archive with BioProject number PRJNA925866.
