# Supplementary material for: Soybean and Cotton Spermosphere Soil Microbiome Shows Dominance of Soilborne Copiotrophs
Source: Microbiol Spectr. 2023 Jun 1;11(4):e00377-23. doi: 10.1128/spectrum.00377-23 (PMC10434258; doi:10.1128/spectrum.00377-23)
Supplement: Supplemental file 4 — Supplemental material. Download spectrum.00377-23-s0004.pdf, PDF file, 0.4 MB [file spectrum.00377-23-s0004.pdf]

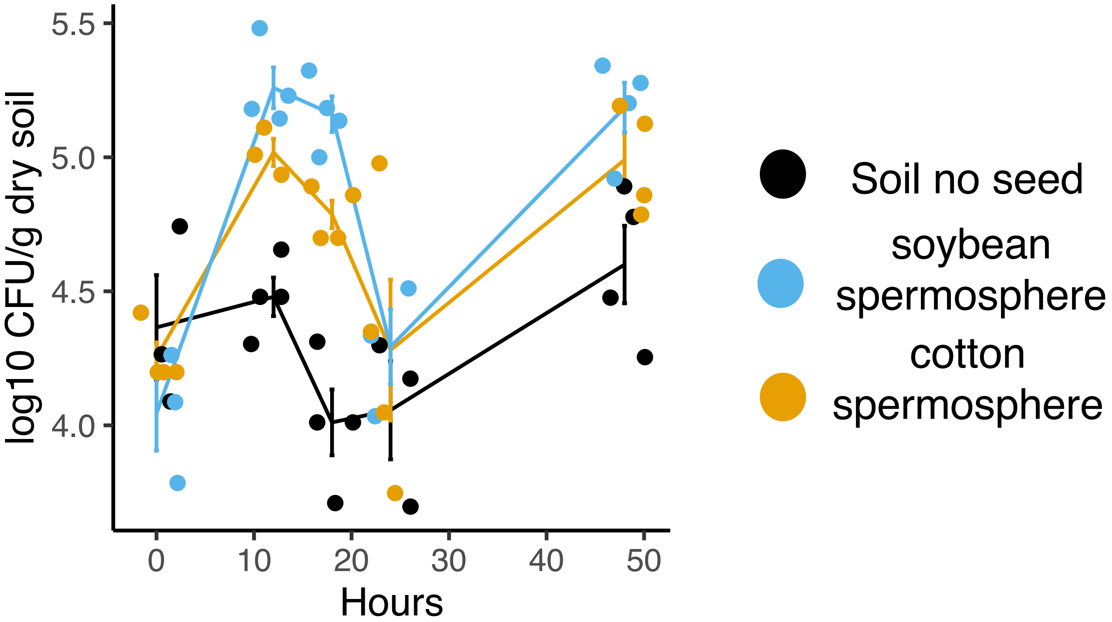

Supplemental Figure 1. Preliminary experiment conducted to demonstrate the effectiveness of the sampling procedure shown in Figure 1. Bacterial populations within spermosphere were greater in soybean or cotton spermosphere soils compared to control soil.

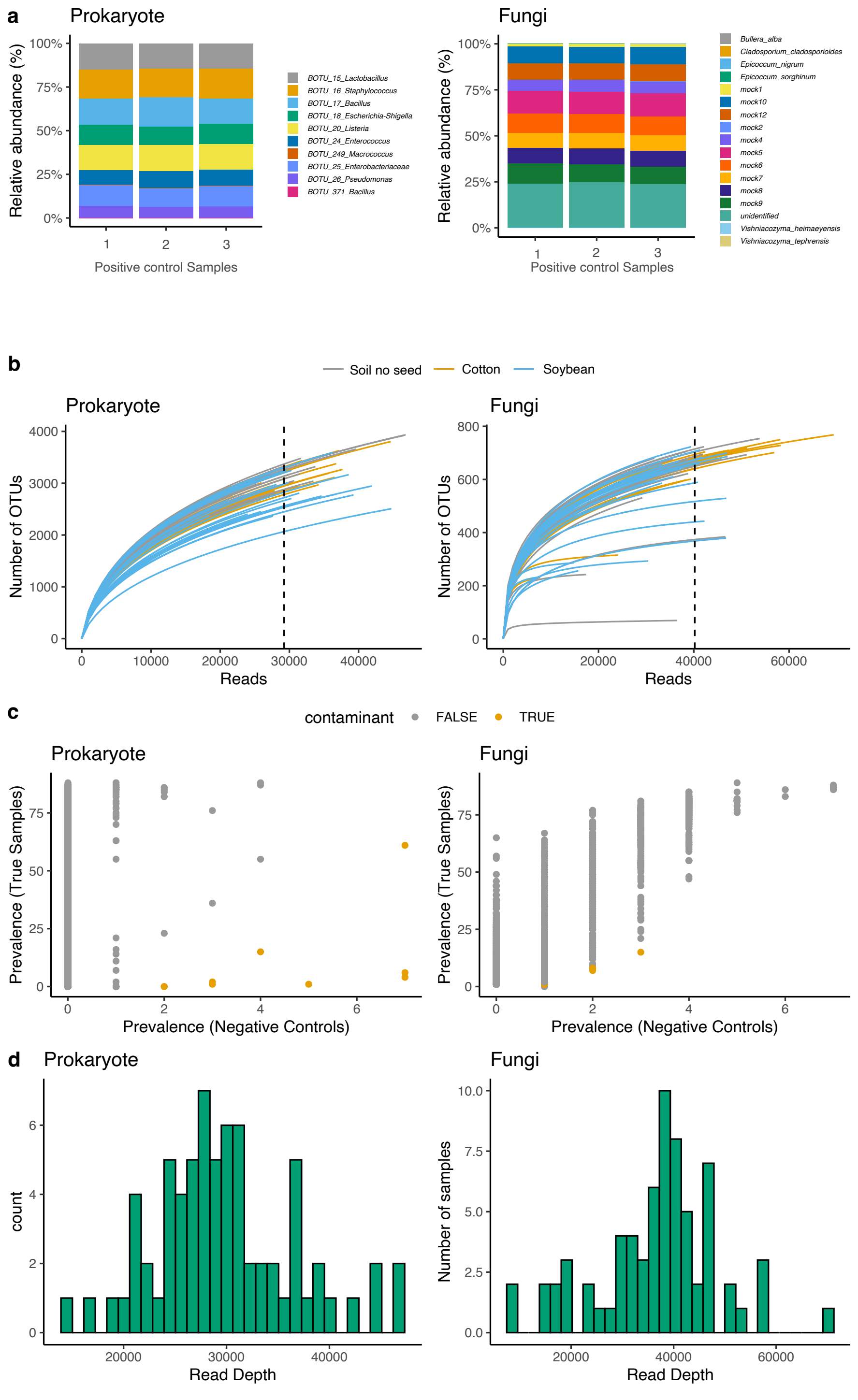

Supplemental Figure 2. Sequencing outputs for prokaryotes (16S) and fungi (ITS). (a) Composition and taxonomic output of the mock community samples. (b) rarefaction curves for prokaryotes and fungi. Dashed lines indicate the median read depth. (c) contaminant filtering based on OTU prevalence in negative control samples. (d) Histogram of read depth per sample.

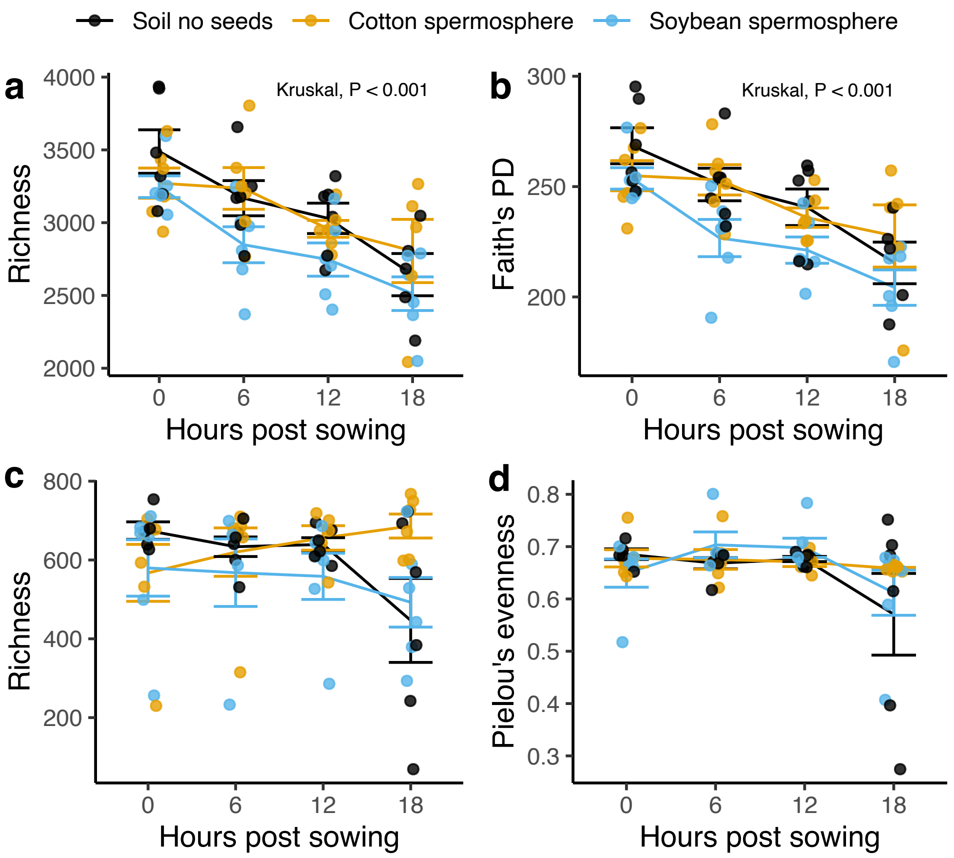

Supplemental Figure 3. Within sample diversity measurements for soybean or cotton spermosphere samples compared to control soil. (a) prokaryote richness significantly dropped over time ($P < 0.001$) consistently across habitats (spermosphere or soil without seed). (b) Similarly, Faith's phylogenetic distance also followed a similar pattern. No consistent differences were observed over time or between habitats for fungal (c) richness or (d) evenness.

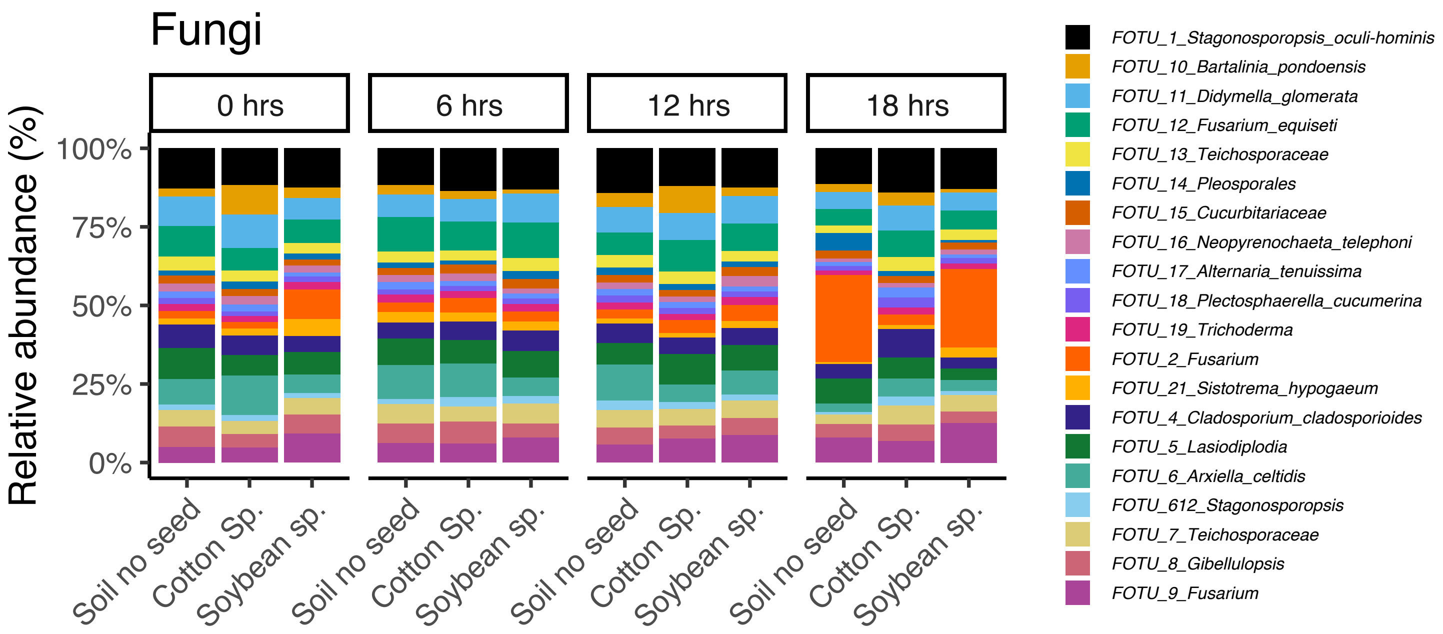

Supplemental Figure 4. Fungal composition of the top 20 most abundant Fungal OTUs in cotton spermosphere soil, soybean spermosphere soil, or control soil.
